# Next generation informatics for big data in precision medicine era

**DOI:** 10.1186/s13040-015-0064-2

**Published:** 2015-11-03

**Authors:** Yuji Zhang, Qian Zhu, Hongfang Liu

**Affiliations:** 1 Division of Biostatistics and Bioinformatics, University of Maryland Greenebaum Cancer Center, Baltimore, USA; 2 Department of Epidemiology and Public Health, University of Maryland School of Medicine, Baltimore, USA; 3 Department of Information Systems, University of Maryland, Baltimore County, Baltimore, MD USA; 4 Department of Health Sciences Research, Mayo Clinic, Rochester, MN USA

## Abstract

The rise of data-intensive biology, advances in informatics technology, and changes in the way health care is delivered has created an compelling opportunity to allow us investigate biomedical questions in the context of “big data” and develop knowledge systems to support precision medicine. To promote such data mining and informatics technology development in precision medicine, we hosted two international informatics workshops in 2014: 1) the first workshop on Data Mining in Biomedical informatics and Healthcare, in conjunction with the 18th Pacific-Asia Conference on Knowledge Discovery and Data Mining (PAKDD 2014), and 2) the first workshop on Translational biomedical and clinical informatics, in conjunction with the 8th International Conference on Systems Biology and the 4th Translational Bioinformatics Conference (ISB/TBC 2014). This thematic issue of BioData Mining presents a series of selected papers from these two international workshops, aiming to address the data mining needs in the informatics field due to the deluge of “big data” generated by next generation biotechnologies such as next generation sequencing, metabolomics, and proteomics, as well as the structured and unstructured biomedical and healthcare data from electronic health records. We are grateful for the BioData Mining’s willingness to produce this forward-looking thematic issue.

## Introduction

Nowadays mining of biological, biomedical, and healthcare data is a fast evolving research area at the intersection between biology and data mining, thanks to unprecedented data deluge in the biomedical research. The informatics field is undergoing an unprecedented era with countless novel breakthroughs aiming to deal with the Big Data mining problems. Taking advantage of these multi-source biomedical data and applying informatics strategies to medicine and care delivery can lead to a next generation precision medicine era [1]. Research that leverages latest multimodal biological measurement technologies with large amounts of healthcare data is in pressing needs. Both workshops, PAKDD 2014 (http://pakdd2014.pakdd.org/) and ISB/TBC 2014 (http://www.aporc.org/ISB/2014/), aim to provide a forum for data miners, informaticians and clinical researchers to share novel findings on their latest investigations in applying informatics techniques to biomedical and healthcare data. The broader context of the workshops comprehends information retrieval, machine learning, natural language processing, and data mining. In particular, papers selected for this thematic issue aim to address the analytical and data mining needs in the informatics field by developing novel methods to mine, summarize and integrate the huge volume and diverse modalities of the structured and unstructured biomedical and healthcare data.

## Research presentations

The five papers selected for this thematic issue are extended versions of the original full-length papers presented at both workshops [MS IDs: 1677560382138468, 1485803967138397, 1425435542146177, 1883484258138342, 5179072941547089]. These papers cover a wide range of analytical topics and their applications to Big Data problems in various informatics research fields.

In the area of natural language processing, Li et al. [MS ID: 1677560382138468] developed an author topic modeling approach to conduct a bibliometric study on tobacco regulatory science (TRS) research. Their results indicated that author topic modeling can help layout the current landscape of TRS and address the issue of research interests reasonably. Furthermore, a network involving authors, topics and words was established for more detailed bibliometric analysis. This network is also useful to grantees and funding administrators in suggesting potential collaborators or identifying those that share common research interests for data harmonization or other purposes.

In the area of information retrieval, Jiang et al. [MS ID: 1883484258138342] presented an *in silico* computational pipeline to mine severe drug-drug interaction (DDI) adverse events (ADE) using semantic web technologies. The approach was applied to a normalized Federal Drug Administration (FDA) Adverse Event Report System (AERS) dataset. A case study was performed on three frequently prescribed cardiovascular drugs: Warfarin, Clopidogrel and Simvastatin. Putative DDI-ADE pairs and their associated outcome codes were extracted. These associations were then validated using ADE datasets from SIDER [2] and Pharmacogenomics Knowledge Base (PharmGKB) [3]. A cross validation strategy was also used using electronic medical records (EMR) data. In total, 601 DDI-ADE pairs for three drugs were identified and validated, of which 61 pairs are in Grade 5, 56 pairs in Grade 4, and 484 pairs in Grade 3. Among 601 pairs, the signals of 59 DDI-ADE pairs were also identified from the EMR data. The proposed approach could be generalized to detect the signals of putative severe ADEs induced by DDIs in other drug domains and would be useful for supporting translational and pharmacovigilance study of severe ADEs. Wang et al. [MS ID: 1485803967138397] constructed a normalized cancer based PGx network (CPN) by integrating cancer orientated PGx information from multiple well known PGx resources including the PharmGKB, the FDA Pharmacogenomic Biomarkers in Drug Labeling, and the Catalog of Published Genome-Wide Association Studies. The CPN has the potential to provide comprehensive cancer specific PGx information and support oncology related research, including cancer based drug discovery and drug repurposing. They demonstrated the capability of the CPN for drug repurposing by conducting two case studies.

In the area of data mining, Tao et al. [MS ID: 1425435542146177] introduced their work on summarizing the Vaccine Adverse Event Reporting System (VAERS) data and representing the vaccine-symptom correlations as well as the metadata of their relations using Resource Description Framework (RDF). They applied network analysis approaches to the RDF data to illustrate a use case of the data. This work can be extended in the future by integrating the data with vaccine information from other sources using RDF linked approach to facilitate more comprehensive analyses. To address the challenge how to establish and characterize the interplay among genes that are altered at different stages in the context of a biological process or functional category, Zhang [MS ID: 5179072941547089] developed a network-based approach to analyzing the differentially expressed genes at different time points by integrating molecular interactions and gene ontology information. The approach was applied to investigate 1α, 25(OH)_2_D_3_ - altered mechanisms in zebrafish embryo development. The results demonstrated that the proposed approach can provide insight on the molecular mechanisms taking place in vertebrate embryo development upon treatment with 1α, 25(OH)_2_D_3_. This approach enables the monitoring of biological processes that can serve as a basis for generating new testable hypotheses. Such network-based integration approach can be easily extended to any temporal- or condition-dependent genomic data analyses.

## Discussions

The primary objective of this thematic issue is to showcase high-quality research in the informatics field, aiming to inspire and educate researchers, practitioners, and students on how to conduct biomedical research in the “Big Data” era. Specifically, both workshops aim to provide research methodologies and tools on managing, analyzing, visualizing, and extracting information from large, diverse, complex, longitudinal, and/or distributed biological, biomedical and healthy data sets. We expect that such next generation informatics approach development will play indispensable roles in mining “big data” that emerges from precision medicine era.
